# Effects of Reducing Sugars on the Structural and Flavor Properties of the Maillard Reaction Products of *Lycium barbarum* Seed Meal

**DOI:** 10.3390/foods12234346

**Published:** 2023-12-01

**Authors:** Tao Chen, Chao-Kun Wei, Tong Li, Hui-Lin Zhang, Zhi-Jing Ni, Mohammad Rizwan Khan, Zhao-Jun Wei

**Affiliations:** 1 School of Food Science and Engineering, Ningxia University, Yinchuan 750021, China; 2 School of Biological Science and Engineering, Collaborative Innovation Center for Food Production and Safety, North Minzu University, Yinchuan 750021, Chinazjwei@hfut.edu.cn (Z.-J.W.); 3 School of Food and Biological Engineering, Hefei University of Technology, Hefei 230009, China; 4 Department of Chemistry, College of Science, King Saud University, Riyadh 11451, Saudi Arabia; mrkhan@ksu.edu.sa

**Keywords:** *Lycium barbarum* seed meal, reducing sugar, Maillard reaction, structural characteristics, flavor

## Abstract

*Lycium barbarum* seed meal contains a variety of bioactive compounds, but the use of *L. barbarum* seed meal in the food industry is rare. This study aimed to evaluate the effect of reducing sugars on the structural and flavor properties of the Maillard reaction products (MRPs) of the *Lycium barbarum* seed meal hydrolysate (LSH). The results showed that the flavors and tastes of the MRPs were affected by reducing sugars. In comparison to oligosaccharides, monosaccharides were more suitable for the development of MRPs with good sensory qualities. The structural characteristics of *L. barbarum* seed meal precursor MRPs were also affected by reducing sugars. The MRPs produced with the participation of monosaccharides had higher ultraviolet absorption and browning than the MRPs produced with oligosaccharides. The molecular weights of the MRPs were found to be 128–500 Da and 500–1000 Da. Compared to the MRPs made from other sugars, xylose-meridian products (X-MRPs) had a stronger meaty flavor. The mellowness and continuity of the MRPs made from monosaccharides were superior to those made from oligosaccharides. The MRPs formed by *L. barbarum* seed meal exhibited the characteristics of umami and meat flavor. MRPs with better flavors may be used to develop new types of seasoning salts.

## 1. Introduction

*Lycium barbarum*, also known as “Wolfberry”, is native to China and is mainly found in Ningxia, Gansu, Qinghai, and Shanxi [1]. *L. barbarum* comprises a number of bioactive substances, including carotenoids, flavonoids, and polysaccharide, and the Ministry of Health of China has identified it as a good source of traditional Chinese medicine [2]. *L. barbarum* deep-processing products mainly include *L. barbarum* pulp, *L. barbarum* beverage, *L. barbarum* wine, and *L. barbarum* seed oil, which are popular with consumers. During the processing and production of *L. barbarum*, a number of by-products are also produced, of which *L. barbarum* pomace from the seeds accounts for a large proportion.

*L. barbarum* seed meal is a by-product of the extraction of the oil from *L. barbarum* seeds. The protein content in *L. barbarum* seed meal is high, and the seed meal protein can be used to obtain functional peptides and amino acids by biological methods. They can then be used to produce good flavors through a Maillard reaction (MR), as well as flavor compounds, including pyrazines and sulfur-containing compounds that can produce characteristic flavors, such as meatiness [3]. *L. barbarum* seed meal protein can also be used for the preparation of food flavors, etc. A small amount of ash is also present in *L. barbarum* seed meal, indicating that it contains small amounts of trace elements and is nutritious. *L. barbarum* seed meal is similar to other plant meals, such as peony seed cake meal and camellia seed cake meal [4,5]. *L. barbarum* seed meal is rich in protein and is frequently used as feed for animals. By giving full play to the active ingredients in the *L. barbarum* seeds, it is possible to reduce the waste of resources during processing and to expand the production chain of *L. barbarum*, which allows for product diversification and also increases the economic value of the product.

MR is a common non-enzymatic browning reaction that occurs during food baking, cooking, and storage, and it is capable of forming different Maillard reaction products (MRPs) [6]. MRs at high temperatures may produce irritating odor substances, such as thiazole, furans, nitrogen-containing compounds, and oxygen-containing compounds [7]. In addition, some toxic substances, such as acrylamide and advanced glycation end products, may be produced. The MRPs formed by *L. barbarum* seed meal are made from the by-products produced during the production and processing of *L. barbarum*. However, carbohydrates and amino compounds with different structures may produce products of different structures and types due to their different reaction mechanisms. Cysteine is considered to be a meat flavor inducer in the formation of MRPs. The addition of cysteine during the MR produces sulfur-containing compounds, which brings the meat flavor to MRPs, and the addition of cysteine can inhibit the pigment and enhance the taste and continuity of MRPs [8]. In recent years, headspace-gas chromatography-ion migration spectroscopy (HS-GC-IMS) and headspace solid-phase microextraction gas chromatography-mass spectrometry (HS-SPME-GC-MS) with high separation capacity and sensitivity have been developed and used to identify flavor compounds and sample quality [9]. Thus, the combination of GC-MS and GC-IMS may reveal a comprehensive picture of flavor changes in food products. However, to our knowledge, this new method has not yet been used to evaluate the flavor characteristics of the MRPs of *L. barbarum* seed meal formed from different reducing sugar types.

In the study, six different reducing sugars (xylose, fructose, arabinose, xylo-oligosaccharides, glucose, and galactose) were used to carry out the MR, and simple systems consisting of reducing sugar, *L. barbarum* seed meal hydrolysate (LSH), and *L*-cysteine were used to evaluate the effect of reducing sugar types on MRPs. We aimed to determine the best flavor of MRPs by measuring the changes in browning pH, color, browning degree, structural characteristics, molecular weight (MW), free amino acids, and flavor substances of different MRPs. In addition, the tastes of MRPs formed from different reducing sugars were evaluated by sensory evaluation through descriptive sensory analysis methods.

## 2. Materials and Methods

### 2.1. Materials and Chemicals

*L. barbarum* seed meal was obtained from Wolfberry Bio-Food Engineering Co., (Ningxia, China). *L*-cysteine, xylose, fructose, galactose, glucose, oligosaccharide, and arabinose were purchased from Wei’s Chemical Reagent Co., Ltd. (Wuhan, China). All the chemicals used were of analytical grade.

### 2.2. Preparation of L. barbarum Seed Meal

*L. barbarum* seed meal was prepared by supercritical carbon dioxide extraction [10]. Briefly, 35 g of fresh *L*. *barbarum* seeds were washed with water to remove impurities, dried at 50–70 °C for about 24–30 h (so that the water content reached 5–15%), and crushed with a grinder to powder (10–13 mesh). The seeds were extracted by a Waters SFE-2 CO_2_ extraction system with a 1 L extraction vessel (Waters Crop., Milford, MA, USA). Pure carbon dioxide (99.99%) was used as the supercritical carrier solvent. The extraction pressure, temperature, time, and CO_2_ flow rate (purity 99.99%) were set as 35 MPa, 40 °C, 90 min, and 1 L/min, respectively. After the oil extracted from the kettle was separated, a large amount of defatted meal was produced, and *L. barbarum* seed meal was formed after freeze-drying for 24 h.

### 2.3. Preparation of L. barbarum Seed Meal Hydrolysate (LSH)

*L. barbarum* seed meal was prepared as a 5% solution, its pH value was adjusted to 8.5, and the amount of alkaline protease added was 3505 U/g. The hydrolysis time was 2.4 h. The pH value of the mixture was then adjusted to 6.5, the dosage of flavored protease was 660 U/g, and the hydrolysis time was 4.2 h. After vacuum freeze-drying, the precursor of the MR of *L. barbarum* seed meal was obtained.

### 2.4. Preparation of MRPs

To prepare MRPs, 1.0 g of LSH, 0.3 g of reducing sugar, and 0.15 g of *L*-cysteine were mixed and incubated at 120 °C for 120 min. Each of the reducing sugars (xylose, fructose, glucose, galactose, arabinose, and xylooligosaccharide) were mixed with LSH and *L*-cysteine. The resulting mixture was adjusted to a concentration of 10%, with a pH of 7.5. The mixture was then placed in a 20 mL ampoule and subjected to a 2 h reaction at 120 °C in an oil bath. The reaction was terminated immediately after completion. The MRPs were obtained by cooling the mixture with ice water, and then they were centrifuged to obtain the supernatant. The supernatant was pre-cooled at −80 °C and then freeze-dried at a low temperature. The resulting products were named based on the sugar used: xylose MRPs (X-MRPs), fructose MRPs (F-MRPs), galactose MRPs (Ga-MRPs), arabinose MRPs (A-MRPs), glucose MRPs (G-MRPs), and xylo-oligosaccharide MRPs (Xo-MRPs). The products were stored at −20 °C.

### 2.5. Determination of Main Components of L. barbarum Seed Meal

The contents of protein and fat were determined through the Kjeldahl method and Soxhlet extraction method, respectively [11]. The ash content was determined according to ISO 2171 (ISO, 2007) [12]. The moisture content was measured according to the direct drying method. The total sugar content was determined by the phenol-sulfuric acid method. The initial moisture content was measured by the moisture rapid tester (Sh10A, Precision Science Instrument Co., Ltd., Shanghai, China). The reducing sugar content was determined by the direct titration method [13].

### 2.6. pH Determination of MRPs

After cooling the aqueous solution of MRPs to room temperature, the terminal pH of the MRPs was determined using a precision pH meter.

### 2.7. Determination of Browning Degree

The browning degree was determined by using a previous method [14]. Briefly, samples were diluted 20- and 50-fold with distilled water, and absorbance was measured at 294 nm (20×) and 420 nm (50×) using a UV/VIS spectrophotometer (UV752, Zhejiang Jueng Equipment Co., Ltd., Shaoxing, China).

### 2.8. UV Absorption Spectroscopy

MRPs were diluted to 1.0 mg/mL before UV absorption spectroscopy measurements were made in the 190–800 nm range, with pure water as a reference.

### 2.9. FI-IR Spectroscopy

The FT-IR spectroscopy of the samples was conducted using the KBr tableting method, and the baseline was collected using KBr as a control [15]. MRPs (25 mg) and KBr (500 mg) were mixed and compacted, and then FI-IR spectroscopy was conducted with an FT-IR spectrometer at wavelengths between 4000 and 400 cm^−1^.

### 2.10. Analysis of Free Amino Acid

The contents of free amino acids were measured by a fully automated amino acid analyzer (L-890003030901, Hitachi, Japan), with an injection volume of 10 μL. The standard amino acid peak area and retention period were used to calculate the amino acid content [16]. Briefly, a sample of 0.1 and 4% 5 mL of sulfosalicylic acid were mixed, and the protein and peptide were precipitated, sonicated for 30 min, and then centrifuged at 10,000× *g* for 20 min. Then, 1.0 mL of the supernatant was added to the vial after passing through a 0.22 μm microporous filter membrane.

### 2.11. Determination of MW Distribution

The MW distribution in the sample was determined by the HPLC method. LC-20A with a Diode array detector and TSK gel 2000 SWXL column (Tosoh, Tokyo, Japan) was used, with a column temperature of 30 °C. The mobile phases were acetonitrile, water, and trifluoroacetic acid at a flow speed of 0.5 mL/min, with a sample size of 10.0 μL. The MW of the standards, cytochrome C, aprotinin, bacitracin, acetyltetrapeptide, and bistem, amino peptide were 12,500, 6500, 1450, 451, and 132 Da, respectively.

### 2.12. GC-MS Analysis of Volatile Compound Composition

Briefly, 5.0 mL of MRPs were added to the headspace of a 20 mL volumetric bottle, and 2 μL of odichlorobenzene (methanol 50 μg/mL) was added as an internal marking, mixed thoroughly, and then sealed. Solid-phase microfibers (75 μm, carboxyl, dimethicone) were adsorbed at 50 °C for 30 min. The ADB-5MS column (30 m × 0.25 mm × 0.25 μm) was used to separate volatile substances. The column temperature was set at 40 °C (2 min), 40 °C–100 °C (2 °C/min), 100 °C–150 °C (4 °C/min), and 150 °C–280 °C (20 °C/min). The mass spectrometer scans were conducted in a scanning range of 35–450 amu and scans were performed at a scan speed of 4.45 amu/s. The retention index (RI) was calculated using n-paraffin (C7–C30) as an injector under the same GC-MS conditions.

### 2.13. GC-IMS Analysis of Volatile Compound Composition

Headspace injection conditions: 0.05 g of MRPs and 2.0 mL of distilled water were added to a 20 mL headspace injection bottle, sealed, and incubated at 60 °C; 500 r/min for 15 min. The sample needle temperature was 65 °C, and sample volume was 200 μL.

GC conditions: column type, 5 ms; column length, 30 m; inner diameter, 0.25 mm; film thickness, 0.25 μm; column temperature, 60 °C; analysis time, 20 min; carrier gas, N_2_ (purity ≥ 99.999%); carrier gas flow rate, 0–2 min, 2 mL/min; 2–20 min, 2 mL/min–100 mL/min.

IMS conditions: drift tube length, 5 cm; tube linear voltage, 400 V/cm; drift gas, N_2_ (purity ≥ 99.999%); flow rate, 150 mL/min; IMS temperature, 45 °C.

### 2.14. Sensory Evaluation

The sensory characteristics of the MRPs were evaluated as reported earlier [4]. The MRP solution (0.5%, *w*/*w*) was dissolved in a umami solution composed of 1.0% (*w*/*v*) sodium glutamate and 0.5% (*w*/*v*) NaCl. Three sensory evaluations were conducted on the umami solution formed by each MRP at room temperature, in dim light, to mask the color differences of MRPs. Ten professionals (4 males and 6 females, aged between 23 and 40) were selected to evaluate the MRPs as an evaluation team after professional training, and the fragrance characteristics of the MRPs were analyzed. Before the sensory evaluation of the six MRPs, the aroma and taste of the MRPs were determined. Eight descriptions, including meaty, umami, bitterness, umami, mouthfeel, caramel, continuity, and overall acceptance, were determined and scored.

Full mouthfeel means the competence of the MRPs to fill the mouth with taste, and continuity means the competence of the MRPs to keep the flavor unchanged in the mouth. The taste of the meat can be described as oiled brisket. When heated, the color of white granulated sugar turned brown, giving it the taste of caramel. Monosodium glutamate was used to describe umami. NaCl solution was used to describe salty taste.

### 2.15. Data Analysis

The results are expressed as mean ± standard deviation (*n* = 3). Data were analyzed by one-way ANOVA, and the differences were analyzed for significance using Duncan’s multiple comparisons using SPSS statistics 20.0 software. *p* < 0.05 was the level of significance.

## 3. Results

### 3.1. Basic Components of L. barbarum Seed Meal

The moisture content of *L. barbarum* seed meal was only 5.42%. After pressing at a high temperature and solvent extraction, the fat content in *L. barbarum* seed meal was 1.48%. This can effectively avoid the generation of bad smells due to excessive fat content during enzymatic hydrolysis. The contents of total sugar and reducing sugar in *L. barbarum* seed meal were 12.36% and 2.52%, respectively. The protein content of *L. barbarum* seed meal was high (22.22%). Therefore, small molecular peptides and amino acids can be hydrolyzed by a protease. There was a small amount of ash in *L. barbarum* seed meal, suggesting that it contained a small amount of trace elements. These results are consistent with the basic components of other plant meals, such as peony seed meal [4].

### 3.2. pH and Browning Degree

**Figure 1:**
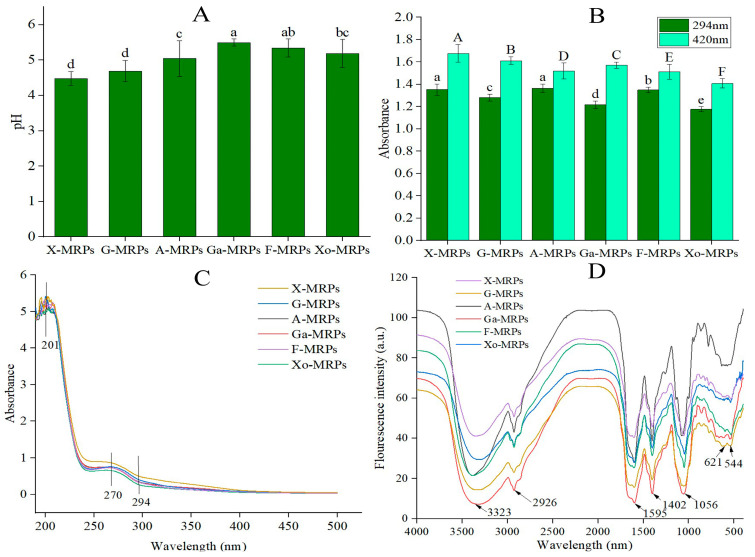
Comparison of the pH values of Maillard reaction products (MRPs) formed by different reducing sugar types, note: Different letters indicate significant difference (*p* < 0.05); (**A**). comparison of the browning intensity of MRPs formed by different reducing sugar types, note: Different lowercase letters indicate significant difference at 294 nm (*p <* 0.05), and different uppercase letters indicate significant difference at 420 nm (*p* < 0.05) (**B**), ultraviolet (UV) absorption spectra within the wavelength range of 100–600 nm (**C**), and Fourier transform infrared (FT-IR) spectra within the frequency range of 4000–500 cm^−1^ (**D**).

The extent of the MR can be evaluated by measuring the degree of pH drop and the intensity of browning [17]. Due to the decrease of amino groups or the coexistence of carbonyl groups and amino groups, the Maillard system produced organic acids during the reaction, reducing the pH value of the solution [18]. As shown in Figure 1A, monosaccharides can be divided into five-carbon sugars and six-carbon sugars, as well as aldoses and ketoses. Different reducing sugars had different MR rates. Different reducing sugars were arranged according to the reaction rate, and the reaction rate was compared with the five-carbon sugar, xylose. The initial pH value of LSH, *L*-cysteine, or reducing sugars (fructose, xylose, glucose, galactose, arabinose, and xylo-oligosaccharides) was 7.5, and after heating and reaction in an oil bath for 2 h, the pH value decreased significantly, and the degree of reduction was as follows: X-MRPs, G-MRPs, A-MRPs, Xo-MRPs, F-MRPs, and Ga-MRPs. The reaction rate of X-MRPs was the fastest, and its PH value decreased most significantly. It was reported that the terminal amino groups of low MW amino acids had high reactivity, increasing the formation of organic acids [19]. As a result, the pH value of the system dropped significantly. The result was consistent with a previous study [20].

The absorbance value A_294_ detects the formation of MR intermediates, and A_420_ detects the formation of brown polymers, such as melanoidin [21]. As shown in Figure 1B, among the MRPs, X-MRPs had the highest absorbance at 294 nm and 420 nm. X-MRPs showed the greatest degree of browning, possibly because xylose is a five-carbon sugar, with a low MW and sterical hindrance; Ga-MRPs had the lowest absorbance at 294 nm; and F-MRPs had the lowest absorbance at 420 nm. Compared with xylo-oligosaccharides, the MRPs formed by monosaccharides had a higher degree of browning, indicating that monosaccharides are more susceptible to browning during the MR. The carbonyl group of xylose can be in full contact with the amino group in the protein hydrolysate to generate MRPs, which are conducive to browning. The color of MRPs changed to brown with the accumulation of melanoidin in the reaction. Aldoses refer to carbohydrates containing an aldehyde group, and ketoses refer to monosaccharides containing a ketone group and their derivatives. Fructose contains a ketone group, which belongs to ketose; both galactose and glucose contain aldehyde groups, which belong to aldehyde sugars. When amino compounds and reducing sugars are heated together, the reaction ability of aldoses is generally stronger than that of ketose. It was found that the sterical hindrance of aldehyde groups was smaller than ketone groups; thus, sugars containing ketone groups may be more likely to undergo MR [22].

### 3.3. UV Spectroscopy and FT-IR Spectroscopy

The UV absorption spectroscopy of F-MRPs, X-MRPs, G-MRPs, Ga-MRPs, A-MRPs, and Xo-MRPs in the range of 190–800 nm was determined. As shown in Figure 1C, since Schiff bases were generated during the reaction, all MRPs exhibited two absorption peaks in the 190–300 nm range. X-MRPs exhibited the largest absorption peak at 201 nm and 270 nm, and the type of sugar was related to the difference in the position of the maximum absorption peak. The type of sugar influenced the absorption of MRPs at 294 nm, consistent with the change in the degree of browning. Different structures of MRPs containing different sulfur-containing substances significantly affected the fluorescence formation pattern [23]. At the beginning of the MR, the condensation of the carbonyl group of reducing sugars with the amino group of proteins formed Schiff bases, which were then converted into an Amadori product. Amadori rearrangement products formed at this stage produced UV absorption at 294 nm [24].

FT-IR oscillates and shifts some molecules by absorbing electromagnetic waves of specific frequencies. It is commonly used to identify functional groups and analyze complex chemical structures [25]. Infrared spectroscopy showed similar positions of absorption peaks (Figure 1D); however, the intensities of the absorption peaks were different in the MR, and the chemical reaction between LSH and sugar caused certain functional groups (such as NH2) to be consumed, resulting in the production of new groups, such as Amadori rearrangement product C=O, Schiff base C=N, pyrazine C-N, and the modification of FT-IR spectroscopy. During the MR, the absorption peak of the hydroxyl group was located in the range of 3001–3556 cm^−1^. When hydroxyl groups formed hydrogen-bonded polymers between molecules, the force constant K decreased; thus, the infrared absorption position moved to a lower wave number (3323 cm^−1^), and OH stretching and NH stretching of hydroxyl groups could be caused by the addition of hydroxyl groups and the consumption of amino groups. There was a small peak of low intensity at around 2926 cm^−1^, indicating the presence of unsaturated C-H bonds. The range of 1309–1697 cm^−1^ was the mixed vibration region of proteins, lipids, and sugars; 1056 cm^−1^ corresponded to the C-O-C stretching of glycosidic bonds; and 710–989 cm^−1^ was the vibration region of carbohydrate isomers [26]. The peak displacements were 1595 cm^−1^ and 1402 cm^−1^, respectively, indicating the formation of Schiff bases.

### 3.4. Free Amino Acid Content and MW Distribution

The levels of free amino acids can affect the sensory performance of MRPs. As shown in Table 1, total free amino acid content decreased after MR (*p* < 0.05). The contents of aspartic acid and glutamic acid produced by X-MRPs were significantly higher than any other sugars (*p* < 0.05). Umami substances can not only produce umami, but they can also increase the mellowness, layering, sustainability, and unique aroma of food. It has been found that the MR can degrade polypeptides (oligopeptides) with umami amino acids to produce umamiamino acids [27]. The content of cysteine in LSH was 0.041 mg/g, while the content of cysteine in all MRPs increased because the content of cysteine in Ga-MRPs was significantly higher than that produced by other reducing sugars (*p* < 0.05) as a result of the addition of *L*-cysteine to the MR. Bitterness is a major indicator of sensory quality of food [19], and the bitter amino acid contents of LSH, F-MRPs, X-MRPs, G-MRPs, Xo-MRPs, A-MRPs, and Ga-MRPs were 6.52 mg/g, 4.70 mg/g, 4.97 mg/g, 4.97 mg/g, 5.09 mg/g, 4.87 mg/g, and 4.88 mg/g, respectively. The contents of isoleucine, leucine, and valine in the MRPs produced from monosaccharides were lower than those produced from oligosaccharides, and the bitter amino acid content of MRPs was significantly lower compared to LSH, indicating that the bitterness of MRPs could be reduced after the occurrence of the MR. It was reported that the MR made it easier for simple sugars to bind to amino acids [28]. During the MR, the reaction of amino acids or small molecule peptides with reducing sugars as reactants could produce aromatic substances, and the addition of *L*-cysteine, on the other hand, added a meaty flavor to the food.

During the MR, the MW distribution of the peptide may also change. As shown in Table 2, the MW of LSH was mainly <128 Da and 128–500 Da, accounting for 24.35% and 51.55%. Compared with LSH, the fraction with MW < 128 Da in MRPs decreased significantly (*p* < 0.05). This is because amino acids and *N*-terminal amino acids of low molecular polypeptides are more active, which easily causes polymerization and cross-linking reactions [29]. In the MR, a large MW product was obtained by cross-linking the reducing sugar with the peptide chain or its degradation products. Among the MRPs produced by different sugars, small peptides or free amino acids were produced due to the thermal decomposition of proteins and reducing sugars during the thermal reaction, compared to LSH, and the content of peptides with an MW distribution of 500–1000 Da was significantly increased (*p* < 0.05). The peptides of 500–1000 Da were reported to have a great impact on the formation of bitterness. The MW distribution of >3000 Da in MRPs increased significantly compared with that in LSH (*p* < 0.05), possibly because melanoidins were produced at the end of the MR, and the production of macromolecular polypeptides in the MR was mainly due to thermal decomposition [30].

### 3.5. GC-MS Analysis of Flavor Components in MRPs

In the six MRPs, a total of 52 volatile compounds were detected, including 33 oxides, 11 sulfides, six nitrogen compounds, and two hydrocarbons (Table S1).

Volatile compound formation was consistent with the reduction of free amino acid content, which was obtained by reacting free amino acids in polypeptide chains or amino groups of free amino acids with carbonyl groups, resulting in a decrease in the content of free amino acids. Sulfur-containing compounds, aldehydes, and lipids have a strong aroma and a low threshold; thus, they contribute greatly to the flavor of the raw material. Sulfur-containing compounds have a significant impact on the overall aroma of different foods [31].

Sulfurous compounds: Sulfur-containing flavor substances were produced in the MR, making the food salty and meaty, with a taste of barbecue. The taste would be very different if there were no sulfur-containing compounds in the product. The thermal decomposition of cysteine or the interaction between carbonyl groups and sulfur-containing amino acids produced sulfur-containing substances. Under the action of cysteine and xylose, cysteine underwent Strecker decomposition to produce H_2_S and NH_3_. In addition, the reaction between furfural and H_2_S can produce 2-furfural mercaptan, 1,4-dicarbonyl compound, and thiophene. In the six MRPs, eleven sulfur-containing substances were detected, including four thiophenes, four thiols, and three sulfur-substituted furans. Sulfur-containing substances had the highest content of X-MRPs, followed by G-MRPs. Thiophene was formed as sugars or carbohydrates in the reaction with the amino acids [32]. Among X-MRPs, the content of thiophene was found to be significantly higher than that of the MRPs produced by the other sugars. 3-Methyl-2-thiophenealdehyde was present in six MRPs, and it is known to give MRPs the flavor of cooked meat. Among X-MRPs, the contents of thiols and thiofurans were higher (*p* < 0.05). The content of 2-methyl-3-furan mercaptan, an important source of sulfur, was significantly higher than other MRPs. This compound has a low odor threshold and is considered one of the main odors of meat [33]. 2-furfuryl mercaptan, 2-methyl-3-furanethiol, and bis(2-furanyl) disulfide were found in cooked foods [34].

Oxygen-containing compounds: 33 oxygenated compounds were identified in the six MRPs, including 14 alcohols, six aldehydes, five furans, four ketones, and four esters. Most of the oxides were produced by the oxidation of fatty acids. Most oxygenated compounds have high flavor thresholds; thus, there was no prominent flavor in MRPs. In the MR of LSH-xylose-*L*-cysteine, oxygen-containing compounds were the most easily produced volatile substances. Furan is an oxygen-containing heterocyclic compound produced by MR and caramelization reaction but also by thermal degradation of the more reducing sugars alone. It has a caramel flavor and can help improve the overall flavor of food [35]. Among the MRPs produced by xylose, the content of furans was significantly higher than those of other sugars, including 5-chloro-n-(furan-2-ylmethyl)-2-nitroaniline and furan. 2-Acetylfuran was not detected in X-MRPs, but it was detected in F-MRPs, Ga-MRPs, and G-MRPs. Fructose, galactose, and glucose are hexose, indicating that the type of sugar had an effect on the formation of furan compounds. A comparison of the MRPs of xylose with xylo-oligosaccharides showed that only X-MRPs contained furans, indicating that adding xylo-oligosaccharides did not promote furan formation. Aldehydes and ketones are largely derived from the degradation and oxidation of fats. X-MRPs had significantly higher levels of aldehydes and ketones than the other five MRPs, including citrone, 4,5-dimethyl-1,3-dioxen-2-one, 2,4-dimethylbenzaldehyde decaldehyde, n-hexanal, nonanaldehyde, benzaldehyde, and 5-methylhexanal. The type and content of aldehydes are related to the antioxidant activity of MRPs [36]. In X-MRPs, n-hexanal and dechardehyde were the main aldehydes; in F-MRPs and A-MRPs, n-hexanal, benzaldehyde, and nonanal were the main aldehydes; in Ga-MRPs, benzaldehyde and nonanal were the main aldehydes; in G-MRPs, the main aldehydes were n-hexanal and benzaldehyde; but in Xo-MRPs, the aldehyde content was low. benzaldehyde is an aromatic aldehyde commonly used in the food industry with a cherry flavor that may come from the Strecker degradation of isoleucine [37,38]. N-hexanal, a main component of vegetables and fruits, with a fruity flavor, may be the result of 2-octene degradation. These substances can enhance the aroma of MRPs. 1-Octen-3-ol was detected in A-MRPs, G-MRPs, and Ga-MRPs. It has an attractive mushroom aroma, anti-spoilage and anti-bacterial activity, and is often regarded as a characteristic aromatic substance for meat foods, such as lamb and chicken. In general, esters help produce a pleasant sea food aroma and increase the freshness of MRPs [39].

Nitrogenous compounds: Six nitrogen compounds were detected in the MRPs, including three pyridine, two pyrazine, and one pyrrole. Pyrazine, an important class of nitrogen-containing heterocycles, usually has the aromas of barbecue, nuts, and burnt foods, formed by the condensation of amino ketones produced by Strecker degradation, and the pyrazine detected in the MRPs were 2,3,5,6-tetramethylpyrazine and 2-methylpyrazine. Only one pyrrole, 2-pyrrolidinemethanol, was detected in the MRPs, and the content of pyrrole in the X-MRPs was much higher than in those of the other five MRPs (*p* < 0.05). A-MRPs had a higher pyrazine content (*p* < 0.05).

### 3.6. GC-IMS Analysis of Flavor Components in MRPs

**Figure 2:**
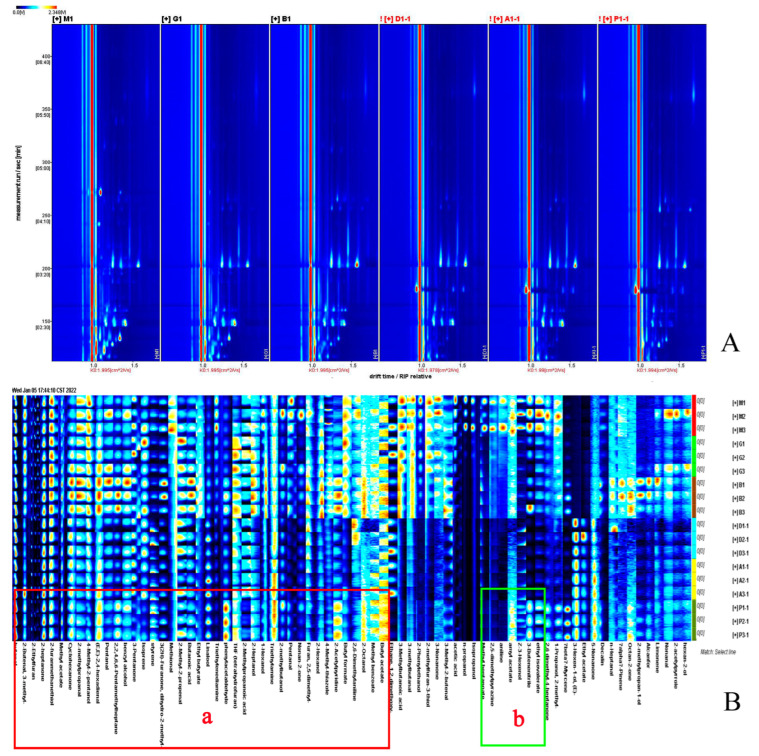
Two-dimensional (2D) GC-IMS spectra of different Maillard reaction products (**A**), gallery plot of the selected signal peak areas obtained with different Maillard reaction products (**B**).

The two-dimensional spectroscopy of the volatile compounds of the six MRPs are shown in Figure 2A. The ordinate in the figure is the retention time, sitting horizontally, labeled as ion migration time. The background is blue, and the red vertical line on the side is the reactive ion peak. The difference of volatile flavor compounds between different samples is mainly reflected in the location, number, intensity, and time of ion peaks. The longer the volatile compound retention time in the migration spectroscopy, the longer the corresponding drift time. The drift time range of organic matter is 1.0–2.0, and the retention time is 100–800 s. White represents low concentrations, red represents high concentrations, and darker colors indicate higher concentrations. Within 100–800 s, some compounds changed from blue to white, indicating that during the MR, some substances disappear, or from white to blue, indicating that some new compounds are produced. There was no further color change after the retention time after 800 s, indicating no migration of flavor compounds and organics after 800 s.

Flavor substances are an important component of MRPs. The volatile compounds in the MRPs were identified using the GC-IMS database after comparing the retention and migration times of the characteristic flavor components. As shown in Table S2, a total of 83 compounds were identified in the six MRPs, including 18 alcohols, 15 aldehydes, 15 esters, 14 hydrocarbons, 10 ketones, eight nitrogenous compounds, and three sulfur-containing compounds.

Aldehydes, esters, and alcohols are the main flavor compounds. Aldehydes have a strong impact on flavor due to the low odor threshold. Alcohol has less of an effect on MRP flavor due to the higher threshold value, but it has a synergistic effect on the overall odor. Phenylacetaldehyde has a honey-like aroma, and it degrades phenylalanine to the corresponding Strecker aldehyde through unsaturated fatty oxidation [40]. Therefore, it is speculated that these unsaturated aldehydes may produce more phenylethylaldehyde. Esters provide fruity, floral, honey, and other odors to the MRPs. The formation of short-chain fatty acids, such as valeric acid and hexanoic acid, is associated with the degradation of fats and amino acids. Normally, it shows a pleasant aroma at low concentrations but an unpleasant taste at high concentrations [41].

### 3.7. Fingerprinting and Content Variation of Volatile Compounds in MRPs

In order to show more specifically and visually the variation pattern of volatile compounds in the products of the MRPs obtained from different reducing sugars, the signal values of all volatile organic compounds in the ion mobility spectroscopy of the MRPs were used to generate flavor substance fingerprints of the MRPs. In Figure 2B, parallel MRP samples of three different sugars are shown vertically (X-MRPs, F-MRPs, G-MRPs, A-MRPs, Ga-MRPs, and Xo-MRPs from top to bottom), and the same volatile organic compounds in MRPs are shown horizontally (the darker the color, the higher the concentration of the substance) [42]. The red boxes in the figure show the substances common to the six MRPs, including 2-methyl-2-propenyl butyraldehyde, 4-methyl-2-pentanol, benzyl alcohol, 2-methylbutanol, 2-butenal, pentanal, linalool, 2-heptanol, 1-hexanol, 2-furanyl mercaptan, 3-pentanone, cyclohexanone, 2-methyl propionaldehyde, methyl acetate, 4-methylthiazole, 2-heptanone, butyl formate, butyl butyrate, benzoic acid methyl acetate, butyl acetate, 3(2H)-furanone, tetrahydrofuran, 2-ethylfuran, furan, 2-acetyl pyridine, isoprene, styrene, 2,2,4,6,6-pentamethylheptane, 3-methylbutyric acid, phenylacetaldehyde, (E,E)-2,4-hexadienal, methoxy, butyric acid, triethylenediamine, butanol concentration, 2-methyl propanal, 2-ethylfuran, 2-methyl propanoic acid, methyl benzoate, 2-heptanone, 4-methyl-2-pentanol, 4-methyl-2-pentanol, 2-butenal, and 2,6-dimethylaniline (Area a). These can be classified as aldehydes, alcohols, thiols, ketones, thiazoles, esters, furans, olefins, acids, and amines. The green boxes are the only substances present in the X-MRPs, including propanol, isopropanol, Z-3-hexenol, and methyl heptanoate (Area b).

### 3.8. Principal Component Analysis (PCA) of Volatile Compounds in MRPs

**Figure 3:**
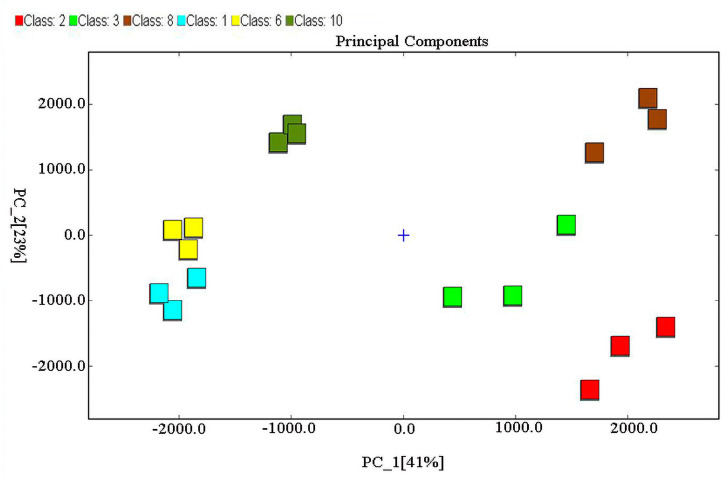
Principal component analysis (PCA) of volatile compounds obtained from MRPs based on the formation of different reducing sugars.

As shown in Figure 3, there were significant differences in the volatile compounds of MRPs formed from different reducing sugars after PCA; two principal components (41% for PC1; 23% for PC2) contributed 64% of the variance. The results suggested that GC-IMS combined with PCA analysis distinguished X-MRPs, Ga-MRPs, and F-MRPs from Xo-MRPs, A-MRPs, and G-MRPs based on the positive values of PC1 and indicated that their aromas differed considerably. On the whole, the scatter distribution of each sample within the group was clustered with each other, showing that better repeatability within groups and sample data was similar. This tended to be equal to the results of the pattern of variation in the GC-IMS 2D profiles of the MRPs (Figure 2A).

### 3.9. Comparison of GC-MS and GC-IMS Results

In order to compare the results of volatile compounds between GC-MS and GC-IMS, the volatile compounds were compared by the number of species. A total of 52 and 83 flavor compounds were identified by GC-MS and GC-IMS respectively. As shown in Table 3, GC-IMS detected more compounds than GC-MS, and both had their own strengths in the classification of volatile compounds. GC-MS excelled in the detection of the compounds containing sulfur, detecting 11 sulfur-containing compounds, but no acids were detected. GC-IMS was better at detecting alcohols, aldehydes, and esters, detecting 18 alcohols, 15 aldehydes, and 11 esters. A total of six substances were detected by both methods, namely 2-pentanone, 1-hexanol, hexanal, nonanal, 2-heptanone, and 2-nonanone, and the difference may be due to the different principle of determination and sensitivity [43]. For example, there were differences in the operating conditions between GC-MS, which uses programmed temperature increases and vacuum conditions, and GC-IMS, which performs its work at a constant temperature and atmospheric pressure.

The headspace gas feed for GC-MS is headspace solid-phase microextraction, in which GC-MS uses headspace solid-phase microextraction sorption, whereas GC-IMS is a simple and rapid method where the headspace gas is injected directly into the detection device. The GC-IMS technique has the advantage of being highly responsive and sensitive [44]. On the other hand, GC-MS is not sensitive to low levels of substances and usually does not detect them. The two methods have a different focus. GC-MS focuses more on the qualitative and quantitative aspects of substances, and GC-IMS presents fingerprinting of main volatile substances, with more of a focus on differentiating samples [45]. Therefore, the analysis of volatile compounds using both methods is able to compensate for their respective limitations and obtain a more comprehensive knowledge of the changing patterns of volatile compounds in the MRPs formed by different reducing sugar types.

### 3.10. Sensory Evaluation

**Figure 4:**
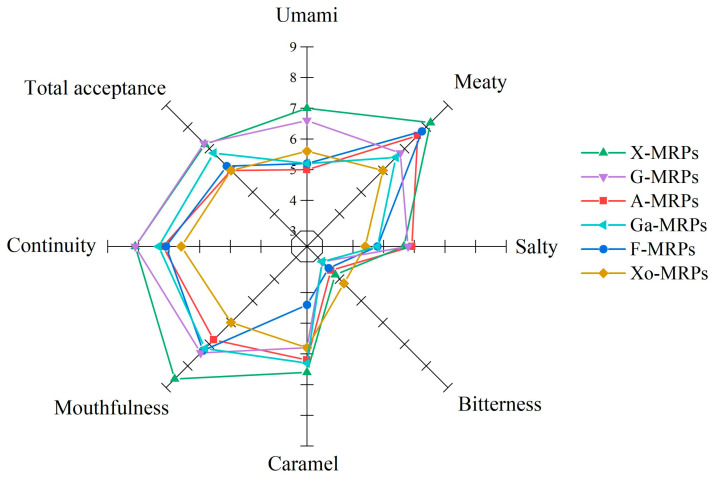
Changes in sensory characteristics as a function of reducing sugar types.

The results of sensory evaluation showed that the types of reducing sugars significantly affected the sensory quality of MRPs (Figure 4), with X-MRPs, F-MRPs, and A-MRPs having significant meat flavor (8.2 ± 0.38, 7.8 ± 0.72, and 7.6 ± 0.41). The meat aroma of the MRPs was mainly derived from the sulfur-containing compounds produced by cysteine during the MR. X-MRPs had a higher caramel aroma, which was associated with their higher furan content. Since the content of bitter amino acids in the MRPs formed by simple sugars was low, their bitterness was lower than that of Xo-MRPs. There was no significant difference in salinity among the six MRPs. X-MRPs scored high in terms of mellowness, durability, and overall acceptance. Results showed that reducing sugars had a significant effect on the sensory quality of MRPs.

## 4. Conclusions

The type of reducing sugar significantly affected the structural and flavor characteristics of MRPs extracted from LSH. FT-IR spectroscopy showed the types and quantities of volatile compound changes in MRPs of *L. barbarum* seed meal following the addition of different sugars for the MR. The UV absorption spectroscopy and browning degree of the MRPs generated by monosaccharides were higher than those produced by xylo-oligosaccharides. The MWs of the MRPs were predominantly 128–500 Da and 500–1000 Da, and sulfur and nitrogenous compounds produced by low MW peptides may produce a meaty taste. Compared to MRPs produced by other sugars, X-MRPs had higher contents of sulfur-containing compounds and umami amino acids, causing X-MRPs to exhibit a stronger meat and umami taste. Compared with xylo-oligosaccharides, monosaccharides were more suitable for the preparation of MRPs with better sensory properties. The mellow feeling and continuity of MRPs produced by monosaccharides were also greater than those produced by Xo-MRPs. Our results showed that there were differences in the flavor and taste of MRPs modified by different reducing sugars. The MRPs modified with different reducing sugars can be used as flavor enhancers, and *L. barbarum* seed meal could be further utilized as an eminent plant protein source for the development of functional foods.

## Figures and Tables

**Table 1:** The contents of free amino acids in *Lycium barbarum* seed meal hydrolysates and Maillard reaction products (MRPs) formed by different reducing sugars (mg/g).

Amino Acid	LSH	X-MRPs	G-MRPs	A-MRPs	Ga-MRPs	F-MRPs	Xo-MRPs
Asp	0.34 ± 0.042 b	0.37 ± 0.035 b	0.31 ± 0.014 d	0.32 ± 0.028 c	0.27 ± 0.035	0.28 ± 0.35 e	0.34 ± 0.070 a
Thr	0.74 ± 0.085 a	0.53 ± 0.071 b	0.46 ± 0.035 de	0.49 ± 0.021 c	0.45 ± 0.021 e	0.43 ± 0.021 f	0.46 ± 0.071 d
Ser	0.34 ± 0.057 c	0.44 ± 0.028 b	0.47 ± 0.092 a	0.33 ± 0.037 d	0.32 ± 0.064 d	0.29 ± 0.028 e	0.43 ± 0.051 b
Glu	1.43 ± 0.13 a	1.42 ± 0.042 d	1.12 ± 0.106 a	1.23 ± 0.071 c	1.25 ± 0.18 c	1.13 ± 0.11 d	1.35 ± 0.13 b
Gly	0.15 ± 0.0071 a	0.11 ± 0.47 d	0.12 ± 0.014 c	0.12 ± 0.42 b	0.092 ± 0.028 e	0.092 ± 0.014 e	0.11 ± 0.014 c
Ala	0.38 ± 0.035 a	0.37 ± 0.014 b	0.34 ± 0.028 c	0.36 ± 0.049 b	0.29 ± 0.064 d	0.29 ± 0.064 d	0.33 ± 0.028 c
Cys	0.041 ± 0.042 g	0.86 ± 0.070 f	1.71 ± 0.042 b	1.47 ± 0.34 d	1.87 ± 0.053 a	0.67 ± 0.099 e	1.57 ± 0.28 c
Val	0.72 ± 0.070 a	0.54 ± 0.099 d	0.57 ± 0.021 c	0.56 ± 0.078 c	0.53 ± 0.12 d	0.47 ± 0.13 e	0.59 ± 0.113 b
Met	0.35 ± 0.092 a	0.26 ± 0.13 bc	0.26 ± 0.078 bc	0.24 ± 0.043 c	0.24 ± 0.21 c	0.21 ± 0.11 d	0.28 ± 0.085 b
Ile	0.66 ± 0.042 a	0.39 ± 0.071 c	0.39 ± 0.0042 cd	0.36 ± 0.12 de	0.33 ± 0.014 ef	0.32 ± 0.30 f	0.47 ± 0.092 b
Leu	1.49 ± 0.0073 a	1.02 ± 0.0073 bc	1.02 ± 0.021 c	0.87 ± 0.20 de	0.91 ± 0.40 d	0.84 ± 0.51 e	1.08 ± 0.091 b
Tyr	0.85 ± 0.13 a	0.86 ± 0.028 a	0.90 ± 0.078 a	0.83 ± 0.17 a	0.78 ± 1.91 a	0.87 ± 0.41 a	0.81 ± 0.042 a
Phe	2.12 ± 0.064 a	1.65 ± 0.021 d	1.67 ± 0.085 cd	1.73 ± 0.18 bc	1.77 ± 0.30 b	1.61 ± 0.54 d	1.54 ± 0.078 e
Lys	0.68 ± 0.0028 a	0.51 ± 0.19 de	0.54 ± 0.035 bcd	0.52 ± 0.071 cde	0.55 ± 0.13 bc	0.58 ± 0.33 b	0.48 ± 0.064 e
His	0.47 ± 0.15 a	0.39 ± 0.38 b	0.39 ± 0.042 b	0.30 ± 0.057 c	0.38 ± 0.11 b	0.33 ± 0.32 c	0.29 ± 0.072 c
Arg	2.00 ± 0.11 a	0.94 ± 0.78 e	1.65 ± 0.76 b	1.58 ± 0.19 b	1.49 ± 0.11 c	1.37 ± 0.42 d	1.32 ± 0.59 d
Pro	0.89 ± 0.26 a	0.55 ± 0.86 bc	0.57 ± 0.12 bc	0.56 ± 0.049 bc	0.53 ± 0.085 c	0.55 ± 0.92 bc	0.58 ± 0.23 b
Total	13.63 ± 0.28 b	11.17 ± 0.96 e	12.50 ± 0.12 c	15.00 ± 0.66 a	12.08 ± 2.67 d	10.34 ± 1.74 f	12.07 ± 0.66 d
EAA	6.75 ± 0.19 a	4.91 ± 0.32 b	4.91 ± 0.027 b	4.77 ± 0.46 d	4.79 ± 0.83 c	4.47 ± 1.15 e	4.91 ± 0.38 b
UAA	1.77 ± 0.13 a	1.78 ± 0.053 a	1.42 ± 0.11 e	1.55 ± 0.068 c	1.52 ± 0.15 d	1.41 ± 0.10 f	1.72 ± 0. 042 b
BAA	6.52 ± 0.15 a	4.97 ± 0.24 c	4.97 ± 0.34 c	4.87 ± 0.57 d	4.88 ± 2.02 d	4.70 ± 1.39 e	5.09 ± 0.16 b
SAA	0.38 ± 0.094 g	1.12 ± 0.16 e	1.98 ± 0.25 b	1.70 ± 0.21 d	2.11 ± 0.15 a	0.88 ± 0.026 f	1.85 ± 0.26 c

Note: Means within different letters were significantly (*p* < 0.05) different on the same line. Abbreviations: X-MRPs, xylose MRPs; F-MRPs, fructose MRPs; Ga-MRPs, galactose MRPs; A-MRPs, arabinose MRPs; G-MRPs, glucose MRPs; Xo-MRPs, xylo-oligosaccharide MRPs; EAA, essential amino acid; UAA, umami amino acids; BAA, bitter amino acids; SAA, sulfur-containing amino acids. EAA = Val + Leu + Ile + Lys + Thr + Phe; UAA = Glu + Asp; BAA = His + Arg + Tyr + Val + Phe + Lys + Leu; SAA = Cys + Met.

**Table 2:** Molecular weight (MW) distribution (%) of *Lycium barbarum* seed meal hydrolysates and Maillard reaction products (MRPs) formed by different reducing sugars.

Sample	MW				
	<128 Da	128–500 Da	500–1000 Da	1000–3000 Da	>3000 Da
LSH	24.35 ± 0.92 a	51.55 ± 0.43 a	2.68 ± 0.24 g	14.04 ± 0.28 c	7.38 ± 0.26 b
X-MRPs	8.33 ± 0.32 f	38.27 ± 0.52 b	36.96 ± 0.36 b	2.48 ± 0.20 e	13.96 ± 0.14 a
G-MRPs	13.33 ± 0.77 c	36.14 ± 0.42 c	32.47 ± 0.27 d	4.04 ± 0.28 d	14.02 ± 0.26 a
A-MRPs	9.12 ± 0.66 d	31.37 ± 0.36 c	25.50 ± 0.37 e	20.02 ± 0.33 a	13.99 ± 0.075 a
Ga-MRPs	9.21 ± 0.41 d	34.74 ± 0.42 bc	24.63 ± 0.25 f	17.33 ± 0.24 b	14.09 ± 0.15 a
F-MRPs	8.56 ± 0.24 e	37.53 ± 0.26 b	37.36 ± 0.42 a	2.53 ± 0.17 e	14.02 ± 0.05 a
Xo-MRPs	18.39 ± 0.91 b	25.64 ± 0.25 d	34.67 ± 0.41 c	7.10 ± 0.25 d	14.20 ± 0.35 a

Note: Means within different letters were significantly (*p* < 0.05) different on the same line.

**Table 3:** The comparison of volatile compounds in Maillard reaction products (MRPs) formed from different reducing sugars by GC-MS and GC-IMS.

Volatile Compounds	GC-MS	GC-IMS
Sulfur-containing compounds	3-Methyl-2-thiophenecarboxaldegyde	2-furanmethanethiol
	5-Methyl-2-thiophenecarboxaldegyde	2-methylfuran-3-thiol
	3-methyl-thiophene	4-Methyl-thiazole
	2-thiophene acetic acid	—
	thioalcohol	—
	2-pentanethiol	—
	2-methyl-3-furanthiol	—
	2-Methyl-3-pentanethiol	—
	Bis(2-furfuryl)disulfide	—
	Bis(2-methyl-3-furyl)disulphide	—
	2-furyl-2-methyl-3-furyl disulfide	—
Nitrogen-containing compounds	Pyridine-N-oxide	2,5-dimethylpyrazine
	3-carboxylic acid	2-ethyl-6-methylpyrazine
	3-1-methylbutyl	2-Acetylpyridine
	2-pyrrolidine methanol	2-acetylpyrrole
	2-methyl-pyrazine	Aniline
	Tetramethylpyrazine	2,6-Dimethylaniline
	—	Triethylenediamine
	—	Triethylamine
Alcohols	Benzyl alcohol	n-propanol
	Phenethyl alcohol	Isopropanol
	1,2-benzenediol	benzyl alcohol
	1-Hexanol	Z-3-Hexenol
	1-pentanol	2-methylbutanol
	1-butanol	Linalool
	1-decanol	1-Propanol
	1-octen-3-ol	2-Phenylethanol
	2,7-dimethyl-1-octanol	2-methylpropan-1-ol
	1,3-pentanediol	2-Heptanol
	Hexaethylene glycol	hexan-2-ol
	2-Hexadecanol	1-Octanol
	Octaethylene glycil	1-Hexanol
	Heptaethylene glycil	1-Hexen-1-ol
	—	1-Octanol
	—	n-Heptanol
	—	2-furanmethanethiol
	—	2-methylfuran-3-thiol
Aldehydes	Hexanal	2-methylpropanal
	Benzaldehyde	2-Butenal
	Decanal	Phenylacetaldehyde
	2,4-dimethylbenzaldehyde	Pentanal
	5-methylhexanal	(E,E)-2,4-hexadienal
	Nonanal	3-methylbutanal
	—	2-Methyl-2-propenal
	—	3-Methyl-2-butenal
	—	Hexanal
	—	Butanal
	—	(E)-2-Pentenal
	—	Nonanal
	—	n-Heptanal
	—	Octanal
	—	(E,E)-2,4-Nonadienal
Ketones	2-heptanone	2-heptanone
	Acetone	2-Pentanone
	2-pentanone	3-Pentanone
	2-Nonanone	Cyclohexanone
	—	2,6-dimethyl-4-heptanone
	—	2-Nonanone
	—	Octan-2-one
	—	5-Nonanone
	—	3-Nonanone
	—	3(2H)-Furanone
Esters	Ethyl laurate	Methyl acetate
	Methyl eicosanoate	Methyl heptanoate
	Isopropyl palmitate	ethyl isovalerate
	Diisooctyl diphosphate	Ethyl butyrate
	—	Butyl formate
	—	butyl butanoate
	—	ethyl heptanoate
	—	Ethyl isopentanoate
	—	amyl acetate
	—	Methyl benzoate
	—	ethyl octanoate
	—	Butyl acetate
	—	Pentyl butyrate
	—	Ethyl acetate
	—	isopentyl acetate
Hydrocarbons	2,4-dimethyl-1-heptene	Triethylamine
	2-pentene	Isoprene
	—	*β*-Myrcene
	—	Styrene
	—	Limonene
	—	α-Pinene
	—	2,2,4,6,6-Pentamethylheptane
Acids	—	acetic acid
	—	3-Methylbutanoic acid
	—	Butanoic acid
	—	2-Methylpropanoic acid
Furan	5-benzofuran ethylamine	THF (tetrahydrofuran)
	2-[(2-Ethoxy-3,4-dimethyl-2-cyclohexen-1-ylidene)methyl]furan	2-ethyl furan
	5-chloro-n-(furan-2-ylmethyl)-2-nitroaniline	Furan
	2-Furfurylthiol	—
	2-Acetylfuran	—

Note: “—” indicates that they were not detected in GC-MS or GC-IMS.

## Data Availability

The data used to support the findings of this study can be made available by the corresponding author upon request.

## Supplementary Materials

The following supporting information can be downloaded at: https://www.mdpi.com/article/10.3390/foods12234346/s1, Table S1: Volatile compounds detected in Maillard reaction products (MRPs) formed by different reducing sugars (ng/g); Table S2: Volatile compounds of Maillard reaction products (MRPs) formed from different reducing sugar types by GC-IMS.

## Abbreviations

A-MRPs, arabinose MRPs; F-MRPs, fructose MRPs; FT-IR, Fourier transform infrared; G-MRPs, glucose MRPs; Ga-MRPs, galactose MRPs; HS-GC-IMS, headspace gas chromatography-ion migration spectroscopy; HS-SPME-GC-MS, headspace solid-phase microextraction gas chromatography-mass spectrometry; LSH, *Lycium barbarum* seed meal hydrolysates; MR, Maillard reaction; MRPs, Maillard reaction products; MW, molecular weight; PCA, principal component analysis; UV, ultraviolet; X-MRPs, xylose MRPs; Xo-MRPs, Xylo-oligosaccharide MRPs.
