# Supplementary material for: Effects of Reducing Sugars on the Structural and Flavor Properties of the Maillard Reaction Products of Lycium barbarum Seed Meal
Source: Foods. 2023 Dec 1;12(23):4346. doi: 10.3390/foods12234346 (PMC10706057; doi:10.3390/foods12234346)
Supplement: Supplementary file 1 [file foods-12-04346-s001.zip › foods-2643023-SI.pdf]

Table S1 Volatile compounds detected in Maillard reaction products (MRPs) formed by different reducing sugars (ng/g)

Count	Compounds	RI[a]	Amounts (ng/g)[b]						Odor description [c]
			X-MRPs	F-MRPs	A-MRPs	Ga-MRPs	G-MRPs	Xo-MRPs	
	**Sulfur-containing compounds**	—	920.94	303.71	390.09	147.61	655.19	93.32	—
	**Thiophene**	—	300.26	162.88	133.82	142.18	283.08	52.95	—
1	3-Methyl-2-thiophenecarboxaldegyde	1128	268.51±12.6a	125.55±1.5c	101.76±0.2d	102.25±0.3d	243.65b	52.95±0.3e	Fatty taste
2	5-Methyl-2-thiophenecarboxaldegyde	1079	14.23±2.0b	27.52±2.0b	25.81±2.1b	39.93±3.9a	7.74±0.5c	ND	—
3	3-methyl-thiophene	795	17.52±2.1b	9.81±1.6c	6.25±0.5cd	ND	26.52±5.2a	ND	Pungent
4	2-thiophene acetic acid	1287	ND	ND	3.45±0.12b	ND	5.17±0.7a	ND	Nutty
	thioalcohol	—	538.24	137.25	224.90	ND	326.78	35.74	—
5	Furfuryl mercaptan	923	207.73±44.4a	137.25±0.53c	211.62±4.4a	ND	189.54±1.22b	35.74±0.94d	Boiled meat
	**Sulfur-containing compounds**	—	920.94	303.71	390.09	147.61	655.19	93.32	—
6	2-pentanethiol	816	12.74±0.38a	ND	13.28±1.8a	7.58±0.52c	11.86±0.06b	ND	Savory
7	2-methyl-3-furanthiol	862	273.22±33.9a	ND	ND	ND	125.38±0.49	ND	Meaty
8	2-Methyl-3-pentanethiol	925	44.55±7.9a	ND	ND	ND	ND	ND	—
	**Sulfur-substituted furans**	—	82.44	3.58	31.37	5.43	45.33	4.63	—
9	Bis(2-furfuryl)disulfide	1679	37.35±3.5a	ND	ND	ND	28.85±0.96b	4.63±0.63c	burned taste
10	Bis(2-methyl-3-furyl)disulphide	1520	15.27±5.9b	3.58±0.4d	21.84±3.5a	5.43±0.68c	16.48±0.49b	ND	Roast beef
11	2-furyl-2-methyl-3-furyl disulfide	1623	29.82±2.8a	ND	9.53±0.8b	ND	ND	ND	Floral
	**Nitrogenous compounds**	—	143.65	70.77	58.65	37.47	110.72	28.59	—
	**pyridine**	—	ND	3.85	2.07	ND	0.2	ND	—

12	Pyridine-N-oxide	1115	ND	ND	ND	ND	0.21±0.04a	ND	umami
13	3-carboxylic acid	1180	ND	2.45±0.27a	2.07±0.2b	ND	ND	ND	—
14	3 - 1-methylbutyl	1342	ND	1.40±0.2a	ND	ND	ND	ND	Fruity
15	2-pyrrolidine methanol	887	143.65±13.8c	66.92±8.1b	28.06±2.3d	37.47±6.3d	103.65±6.5a	28.59±0.59d	Fruity
	**Pyrazines**	ND	ND	ND	28.52	ND	6.86	ND	—
16	2-methyl-pyrazine	854	ND	ND	28.52±2.1a	ND	ND	ND	Roasted
17	Tetramethylpyrazine	1086	ND	ND	ND	ND	6.86±0.03a	ND	—
	**Oxygenated compounds**	—	2325.09	1935.51	1596.32	966.6	1966.54	333.88	—
	**furan**	—	576.46	64.52	211.63	22.67	406.21	39.89	—
18	5-benzofuran ethylamine	1204	76.65±14.3a	44.58±0.4c	ND	ND	58.38±0.93b	ND	Savory
19	2-[(2-Ethoxy-3,4-dimethyl-2-cyclo hexen-1-ylidene)methyl]furan	1623	37.57±2.4a	ND	ND	13.09±0.9c	25.68±0.83b	ND	—
20	5-chloro-n-(furan-2-ylmethyl) - 2-nitroaniline	1263	254.53±11.1a	ND	ND	ND	104.78±0.49b	39.89±0.69c	—
21	2-Furfurylthiol	916	207.7±44.4a	ND	211.6±4.4a	ND	201.43±0.39a	ND	Cofffee
22	2-Acetylfuran	1032	ND	19.94±3.5a	ND	9.58±1.3b	15.94±1.2a	ND	Balsamic, Cocoa,
	**Ketones**	—	11.29	18.50	8.17	14.08	ND	ND	—
23	2-heptanone	839	11.29±0.46a	10.27±0.72a	4.06±0.32b	6.25±0.5b	ND	ND	Nutty, Spice
24	Acetone	794	ND	ND	2.33±0.28a	ND	ND	ND	—
25	2-pentanone	934	ND	ND	1.78±0.15b	7.83±0.5a	ND	ND	Pungent
26	2-Nonanone	1090	ND	8.23±0.95a	ND	ND	ND	ND	Fragrant
	**Alcohols**	—	906.52	1325.92	778.28	670.67	7605.7	196.51	—
27	Benzyl alcohol	1275	27.72±11.15a	4.87±0.27b	ND	ND	5.70±0.75b	ND	Roasted Bread
28	Phenethyl alcohol	1373	18.56±7.83b	4.68± 1.94c	ND	ND	145.13±11.95a	ND	—

29	1,2-benzenediol	1753	54.47±3.56e	254.43±31.26c	345.26±36.26b	42.55±57.17a	33.17±4.45e	97.66±11.24d	—
30	1-hexanol	991	34.14±2.13bc	97.36±24.63a	47.83±5.24b	42.53±7.26b	15.25±5.27cd	11.48±3.26d	Grass, Herb
31	1-pentanol	734	ND	76.18±5.17a	ND	ND	ND	ND	Balsamic
32	1-butanol	714	ND	358.02±19.93a	ND	ND	ND	ND	Fruity
33	1-decanol	1081	45.74±1.47a	3.69±0.77c	4.09±0.58c	ND	ND	15.59±1.86b	Fat, Oil
34	1-octen-3-ol	1161	ND	ND	49.53±8.36c	67.77±13.34b	74.17±5.13a	ND	—
35	2,7-dimethyl -1-octanol	1012	ND	ND	11.62±1.49a	ND	ND	ND	—
36	1,3-pentanediol	854	251.16±40.83b	509.63±39.13a	248.53±30.23b	512.08±37.83a	332.35±17.47ab	53.63±11.34c	Mushroom
37	Hexaethylene glycol	2048	19.33±2.27a	ND	ND	ND	ND	ND	fragrance
38	2-Hexadecanol	1793	36.64±20.72a	5.56±1.67cd	7.97± 1.95b	5.74±0.22bc	ND	18.15±1.73b	Rose
39	Octaethylene glycil	1566	ND	7.43±1.58b	64.45±4.41a	ND	ND	ND	—
40	Heptaethylene glycil	1542	418.76±39.48a	4.07±0.89b	ND	ND	ND	ND	—
	**Aldehydes**	—	830.82	526.57	598.24	259.18	954.56	98.48	—
41	Hexanal	872	549.52±45.85a	190.54±21.06b	257.69±37.42b	ND	251.55±8.28b	94.78±5.38c	Apple, Fat
42	Benzaldehyde	873	87.63±14.68a	132.44±45.28a	151.66±16.23ab	118.58±26.42a	211.33±21.17a	ND	Bitterness
43	Nonanal	1105	34.47±3.72b	144.93±19.16a	140.14±15.96a	116.48±18.17a	0.36±0.19b	1.19±0.63b	Lemon,Floral

44	Decanal	1200	113.96±10.52a	19.48±1.55c	48.75±1.11b	19.49±4.12c	0.93±0.12d	ND	Citrus , orange
45	2,4-dimethylbenzaldehyde	1528	28.47±2.12b	5.54±0.48b	ND	4.63±0.98b	385.67±34.84a	ND	—
46	5-methylhexanal	1034	16.77±1.28c	33.66±4.08b	ND	ND	104.72±6.26a	2.51±0.54d	Fruity
	**Esters**	—	140.94	18.71	47.87	56.72	27.49	ND	—
47	Ethyl laurate	1590	49.35±2.1a	ND	ND	36.47±1.6b	ND	ND	Aromatic
48	Methyl eicosanoate	1705	45.82±2.6a	9.38±1.1b	ND	ND	ND	ND	Almonds
49	Isopropyl palmitate	1744	45.77±9.3a	ND	ND	ND	ND	ND	Bitter, Cocoa
50	Diisooctyl diphosphate	1012	ND	9.33±0.3d	47.87±3.4b	20.25±1.1c	27.49±1.7a	ND	—
	**Hydrocarbon**	ND	ND	8.23	12.18	ND	ND	ND	—
51	2,4-dimethyl-1-heptene	836	ND	8.23±0.5a	—	ND	ND	ND	—
52	2-pentene	722	ND	ND	12.18±1.0a	ND	ND	ND	orange, honey

a

RI , retention index determined using the n-alkanes C7-C30 on DB-5MS column (30 m × 0.25 mm × 0.25 μm).

b

Means within different letters were significantly ($p < 0.05$) different on the same line.

c

Odor descriptions were from FEMA database and literature (Wang et al.,2020; Qinzhu et al.,2018; Xie et al., 2022; Feng et al., 2018; Chun et al.,2020; Liu et al.,2022; Habinshuti et al., 2022).

Note: ND and “—” indicated not detected.

Table S2 Volatile compounds of Maillard reaction products (MRPs) formed from different reducing sugar types by GC-IMS

Compound	CAS#	Formula	Peak area variation						Odor description
			X-MRPs	F-MRPs	G-MRPs	A-MRPs	Ga-MRPs	Xo-MRPs	
2-methylpropanal	78-84-2	C_4H_8O	830.76	268.47	501.364	330.25	330.25	501.425	—
2-Butenal	107-86-8	C_5H_8O	466.20	211.20	150.65	501.36	478.23	355.42	—
Pentanal	110-62-3	$C_5H_{10}O$	435.36	532.16	466.20	272.35	243.98	524.31	Pungent
Phenylacetaldehyde	122-78-1	C_8H_8O	256.32	ND	435.36	ND	435.36	ND	Almond
(E,E)-2,4-hexadienal	142-83-6	C_6H_8O	ND	510.26	453.63	302.13	202.28	265.94	Nutty
3-methylbutanal	590-86-3	$C_5H_{10}O$	210.35	374.52	390.10	76.82	374.52	86.48	—
2-Methyl-2-propenal	78-85-3	C_4H_6O	115.36	ND	ND	210.35	ND	317.29	—
3-Methyl-2-butenal	107-86-8	C_5H_8O	653.24	115.36	210.3	139.87	410.35	104.23	—
Hexanal	66-25-1	$C_6H_{12}O$	720.16	607.53	246.87	562.39	653.24	ND	Floral
Butanal	123-72-8	C_4H_8O	289.85	102.37	225.68	ND	825.43	542.15	Creamy
(E)-2-Pentenal	157-68-70	C_5H_8O	ND	ND	320.47	ND	745.36	258.46	—
Nonanal	124-19-6	$C_9H_{18}O$	ND	ND	58.71	48.581	110.86	ND	Fatty, green
n-Heptanal	111-71-7	$C_7H_{14}O$	475.25	352.16	142.57	ND	ND	265.28	—
Octanal	124-13-0	$C_8H_{16}O$	420.16	226.12	ND	ND	ND	56.67	—
(E,E)-2,4-Nonadienal	591-08-72	$C_9H_{14}O$	1076.91	ND	ND	818.39	ND	ND	Tobacco
n-propanol	71-23-8	C3H8O	1706.02	1523.88	325.42	709.22	280.44	643.26	Pungent
Isopropanol	67-630	C_3H_8O	1522.78	1563.77	539.40	128.04	140.82	80.64	—
benzyl alcohol	100-51-6	C_7H_8O	3792.36	ND	ND	ND	ND	87.62	Aromatic
Z-3-Hexenol	928-96-1	$C_6H_{12}O$	ND	984.97	1253.54	619.05	734.69	ND	Grass
2-methylbutanol	137-32-6	$C_5H_{12}O$	2599.95	ND	ND	ND	3291.32	ND	—
Linalool	78-70-6	$C_{10}H_{18}O$	ND	2513.86	436.85	2875.21	2837.20	ND	Fruity
1-Propanol	78-83-1	$C_4H_{10}O$	258.34	ND	356.28	88.11	140.11	356.32	Alcohol, Candy

2-Phenylethanol	60-12-8	$C_8H_{10}O$	ND	352.80	753.54	ND	ND	256.38	—
2-methylpropan-1-ol	78-83-1	$C_4H_{10}O$	417.20	541.38	346.52	468.52	817.53	103.59	—
2-Heptanol	543-49-7	$C_7H_{16}O$	652.34	234.71	541.20	356.24	457.12	ND	Citrus, Fried
hexan-2-ol	626-93-7	$C_6H_{14}O$	300.87	ND	ND	247.31	425.38	857.68	Mint, Cool
1-Octanol	111-87-5	$C_8H_{18}O$	790.15	ND	342.24	142.68	356.96	238.88	Lemon
1-Hexanol	626-93-7	$C_6H_{14}O$	225.32	ND	412.53	356.28	732.23	175.43	Flower
1-Hexen-1-ol	928-97-2	$C_6H_{12}O$	125.39	2463.231	476.53	ND	576.26	ND	—
1-Octanol	123-96-6	$C_8H_{18}O$	780.65	953.96	258.49	1587.62	67.18	81.43	—
n-Heptanol	111-70-6	$C_7H_{16}O$	ND	860.86	485.22	1897.57	82.62	991.62	Aromatic
2-furanmethanethiol	98-02-2	C_5H_6OS	795.38	ND	605.67	ND	48.58	253.12	Coffee, Roasted Meat
2-methylfuran-3-thiol	55764-25-5	C_5H_6OS	812.64	ND	3199.68	2413.15	17.021	ND	—
4-Methyl-thiazole	693-95-8	C_4H_5NS	1253.59	338.54	ND	6222.95	3451.13	64.05	—
2-heptanone	110-43-0	$C_7H_{14}O$	3166.50	2808.49	15.186	2665.34	ND	1019.87	—
2-Pentanone	565-61-7	$C_6H_{12}O$	ND	ND	ND	5079.44	32.831	ND	Fruit, Pungent
3-Pentanone	96-22-0	$C_5H_{10}O$	132.89	44.68	368.47	69.40	79.28	199.01	Grass
Cyclohexanone	108-94-1	$C_6H_{10}O$	88.48	180.82	14.19	ND	245.78	67.18	—
2,6-dimethyl-4-heptanone	108-83-8	$C_9H_{18}O$	553.34	328.27	258.36	ND	ND	82.62	Fruit
2-Nonanone	925-78-0	$C_9H_{18}O$	732.47	524.69	321.72	626.04	1463.23	48.58	—
Octan-2-one	111-13-7	$C_8H_{16}O$	895.36	ND	ND	564.74	953.96	17.02	—
5-Nonanone	502-56-7	$C_9H_{18}O$	742.01	2837.20	ND	795.86	860.86	ND	Grilled
3-Nonanone	925-78-0	$C_9H_{18}O$	335.82	436.21	199.01	245.41	ND	1551.04	—
3(2H)-Furanone	3511-31-7	$C_5H_8O_2$	ND	ND	67.18	2821.75	ND	32.83	Grass
THF (tetrahydrofuran)	18039-90-2	C_4H_8O	113.96	315.39	82.62	1436.64	338.54	79.28	Mint
2-ethyl furan	3208-16-0	C_6H_8O	28.47	ND	48.58	45.77	ND	245.78	Burnt
Furan	625-86-5	C_6H_8O	48.77	2649.20	56.02	60.95	289.67	242.69	Caramel

2,5-dimethyl-4-hydroxy-3(2H)-furanone	3658-77-3	$C_6H_8O_3$	140.94	ND	ND	752.34	415.54	369.58	Fruity,Caramel
Methyl acetate	79-20-9	$C_3H_6O_2$	189.65	480.61	1251.04	45.82	460.21	120.39	—
Methyl heptanoate	106-73-0	$C_8H_{16}O_2$	ND	ND	542.13	672.51	231.24	100.78	Slightly
ethyl isovalerate	108-64-5	$C_7H_{14}O_2$	ND	1330.34	986.35	250.35	124.36	ND	—
Ethyl butyrate	105-54-4	$C_6H_{12}O_2$	247.32	637.29	ND	113.96	ND	1810.47	—
Butyl formate	592-84-7	$C_5H_{10}O_2$	512.34	342.15	225.39	473.52	426.39	573.03	Fruity
butyl butanoate	109-21-7	$C_8H_{16}O_2$	352.71	ND	257.16	421.58	353.09	504.20	Bread
ethyl heptanoate	106-30-9	$C_9H_{18}O_2$	428.50	ND	325.46	785.69	ND	940.23	—
Ethyl isopentanoate	108-64-5	$C_7H_{14}O_2$	ND	245.36	176.80	ND	107.56	ND	Floral, Green Apple
amyl acetate	628-63-7	$C_7H_{14}O_2$	574.23	258.65	ND	104.56	986.5	ND	Balsamic aroma
Methyl benzoate	93-58-3	$C_8H_8O_2$	936.25	ND	457.96	783.24	ND	540.13	Fruity
ethyl octanoate	106-32-1	$C_{10}H_{20}O_2$	356.28	421.01	852.42	754.32	201.54	320.15	Grilled
Butyl acetate	123-86-4	$C_6H_{12}O_2$	ND	ND	174.00	ND	ND	85.63	Fruity
Pentyl butyrate	540-18-1	$C_9H_{18}O_2$	452.16	296.15	223.14	632.15	ND	65.32	—
Ethyl acetate	141-78-6	$C_4H_8O_2$	75.85	152.39	55.65	254.63	410.96	360.12	Apple, Banana
isopentyl acetate	123-92-2	$C_7H_{14}O_2$	53.28	ND	ND	ND	ND	ND	Fruity
2,5-dimethylpyrazine	123-32-0	$C_6H_8N_2$	223.30	392.31	ND	290.65	470.97	377.53	Fried flower
2-ethyl-6-methylpyrazine	13925-03-6	$C_7H_{10}N_2$	ND	535.39	ND	311.02	425.89	324.59	Nutty
2-Acetylpyridine	1122-62-9	C_7H_7NO	101.62	137.22	ND	490.23	54.09	75.68	—
2-acetylpyrrole	1072-83-9	C_6H_7NO	324.15	ND	2139.82	ND	504.27	ND	Bread, Cocoa
Aniline	62-53-3	C_6H_7N	315.39	509.21	406.6	2485.04	20.12	704.78	Stimulating
2,6-Dimethylaniline	87-62-7	$C_8H_{11}N$	ND	654.35	350.66	311.10	ND	338.54	—
Triethylenediamine	280-57-9	$C_6H_{12}N_2$	ND	1330.43	ND	ND	176.80	993.80	—
Triethylamine	121-44-8	$C_6H_{15}N$	ND	ND	1974.53	858.17	185.93	979.22	—

Isoprene	78-79-5	C_5H_8	1231.45	258.96	372.32	107.52	101.08	225.85	—
β-Myrcene	123-35-3	$C_{10}H_{16}$	ND	ND	518.20	336.75	ND	ND	—
Styrene	100-42-5	C_8H_8	90.13	ND	ND	102.56	247.15	651.02	Toasted
Limonene	138-86-3	$C_{10}H_{16}$	463.25	ND	1020.45	ND	1210.53	327.89	—
α-Pinene	80-56-8	$C_{10}H_{16}$	147.69	514.10	ND	312.50	ND	451.78	Nutty
2,2,4,6,6-Pentamethylheptane	13475-82-6	$C_{12}H_{26}$	463.35	ND	465.38	ND	120.36	338.54	Fruity
acetic acid	64-19-7	$C_2H_4O_2$	ND	743.486	ND	634.29	ND	ND	Stimulating
3-Methylbutanoic acid	503-74-2	$C_5H_{10}O_2$	498.63	223.30	165.37	358.17	120.98	ND	—
Methional	3268-49-3	C_4H_8OS	242.36	197.93	85.63	ND	369.85	ND	Sulphur
Butanoic acid	107-92-6	$C_4H_8O_2$	ND	247.8	175.68	569.75	471.30	410.35	Stimulating
2-Methylpropanoic acid	79-31-2	$C_4H_8O_2$	105.29	ND	ND	849.33	ND	652.31	—
3-Butenenitrile	109-75-1	C_4H_5N	ND	353.09	504.20	404.91	ND	780.22	—

Note: “ND” and “—” indicated not detected.
